# The Cross-Entropy Based Multi-Filter Ensemble Method for Gene Selection

**DOI:** 10.3390/genes9050258

**Published:** 2018-05-10

**Authors:** Yingqiang Sun, Chengbo Lu, Xiaobo Li

**Affiliations:** 1 School of Information Science and Engineering, Ningbo University, Ningbo 315000, China; 18363623303@163.com; 2 College of Engineering, Lishui University, Lishui 323000, China; lu.chengbo@aliyun.com

**Keywords:** cross-entropy, multi-filter, gene expression profile, ensemble method, gene selection

## Abstract

The gene expression profile has the characteristics of a high dimension, low sample, and continuous type, and it is a great challenge to use gene expression profile data for the classification of tumor samples. This paper proposes a cross-entropy based multi-filter ensemble (CEMFE) method for microarray data classification. Firstly, multiple filters are used to select the microarray data in order to obtain a plurality of the pre-selected feature subsets with a different classification ability. The top *N* genes with the highest rank of each subset are integrated so as to form a new data set. Secondly, the cross-entropy algorithm is used to remove the redundant data in the data set. Finally, the wrapper method, which is based on forward feature selection, is used to select the best feature subset. The experimental results show that the proposed method is more efficient than other gene selection methods and that it can achieve a higher classification accuracy under fewer characteristic genes.

## 1. Introduction

With the promotion of large-scale gene expression profiles, DNA chips can be used to obtain the expression level of thousands of genes in tissue samples, at the same time, in one experiment. The accurate classification of a tumor subtype at the molecular level is of great significance to the diagnosis and treatment of the tumor. The tumor gene expression profile data usually has a small sample size and high-dimensional feature space [1,2,3,4]. There is plenty of redundancy and noise data in the original data set, and thus the use of the feature selection method for classification can not only reduce the computational time, but it can also improve the classification accuracy [5,6]. Each sample in the data set records the level of expression of all of the measurable genes in the tissue sample, whereas only a few genes are actually related to the sample classification. Knowing how to select a group of genes that are critical to the classification of the sample is a key factor in establishing an effective classification model [7].

The gene selection consists of selecting a subset of genes from all of the attributes of the gene expression profile data [8], and the obtained genes have a strong ability to recognize the disease [9]. There are, in general, two approaches to gene selection, namely, filter [10] and wrappers [11]. The filter approach is based on the characteristics of the data itself for feature selection, and it does not depend on the classification algorithm to predict the selected subset [12,13]. The filter methods can be divided into two groups [14,15,16,17], namely, univariate and multivariate. The univariate methods measure the relationship of a single feature, with respect to a single evaluation criterion. In these methods, the dependencies between features play no role in the feature selection process. Methods such as the signal-to-noise ratio (SNR) [18], *t*-statistics (TS) [19], F-test (FT) [20], and Pearson correlation coefficient (PC) [21] have been shown to be effective for measuring the discriminative power of genes. Unlike the univariate filter methods, the multivariate methods also consider the relationship between the features. This difference makes the multivariate methods relatively slower than their univariate counterparts. Well-known multivariate filter methods include correlation based feature selection (CFS) [22], minimum redundancy maximum relevance (mRMR) [23], and fast correlation based filter (FCBF) [24]. The filter approach is entirely based on the individual vector data for the feature selection, and the final subset evaluation criteria are independent of the classifier. The wrapper method selects a feature subset by some learning algorithms [25,26,27], and then evaluates it through the classifiers [28]. The evaluated data set can be selected and evaluated again, until the optimal feature subset is selected. Different learning methods have different feature subset evaluation criteria, some are based on an intelligent learning algorithm, some are based on a biological significance, and the others are based on its search space. However, the wrapper method is computationally slow and expensive [29]. In summary, the wrapper feature selection methods mainly select the final feature subset with the evaluation criteria of each learning algorithm. 

The cross-entropy method is a new type of stochastic optimization algorithm that has emerged in recent years. It was first used to simulate low probability events and, later, it was extended to solve optimization problems [30,31,32,33]. The method has simple control parameters and a strong robustness. The cross-entropy is gradually applied to the field of bioinformatics, with its unique advantages, and has achieved some good results in the selection of the tumor feature genes. Su et al. [34] utilizes cross-entropy methods to deal with genes and gene pairs selection questions. Lin et al. [35] uses a cross-entropy Monte Carlo method for messenger RNA (mRNA) and microRNA studies problem. Bala et al. [36] uses mutual information in order to sort gene sets and uses the cross-entropy method to select the feature genes in descending order of the ranking results. In addition, this method is also applied to some practical problems in continuous multi-objective optimization and machine learning.

It is well known that many feature selection methods are very sensitive to data perturbations and lead to an instability of the selected classification models [37,38,39]. In order to select accurate classification subsets, many researchers have proposed the ensemble feature selection algorithm [40]. It is carried out by means of weighting several weak or base classifiers, and by combining them in order to obtain a classifier that outperforms each of the other classifiers. The ensemble methods can not only improve the classification accuracy and they can but also achieve the purpose of reducing dimension.

In view of the characteristics of the tumor gene expression data, and in order to obtain the highest possible sample classification rate and to reduce the time complexity of the algorithm, using as few as possible information genes, a cross-entropy based multi-filter ensemble (CEMFE) gene selection algorithm is proposed in this paper, which can select the feature subset with the best classification performance, using the wrapper method, which is based on the forward feature selection, with the accuracy as the criterion.

## 2. Materials and Methods

### 2.1. Datasets

To validate the performance of the proposed algorithm, five public biological datasets were used in this paper, including the Colon, Prostate, Leukemia, Lymphoma, and Lung datasets, which were downloaded [41]. Table 1 gives the detailed information of the five data sets.

### 2.2. Filtering Process

#### 2.2.1. Signal-to-Noise Ratio

(1) $$SNR=\frac{\left|\mu_{g^{+}}-\mu_{g^{-}}\right|}{\delta_{g^{+}}+\delta_{g^{-}}}$$

where *SNR* is the signal-to-noise ratio of gene *g*, $\mu_{g^{+}}$, $\mu_{g^{-}}$, is the mean value of expression level in different sample classes, and $\delta_{g^{+}}$, $\delta_{g^{-}}$ is the standard deviation of the expression level. The signal-to-noise ratio of each gene is calculated, and the genes are sorted from high to low [42].

#### 2.2.2. *t-*Statistic

(2) $$t_{j}=\frac{\bar{x_{2j}}-\bar{x_{1j}}}{\sqrt{s_{1j}{}^{2}/n_{1}+s_{2j}{}^{2}/n_{2}}}$$

$\bar{x_{1j}}$ and $\bar{x_{2j}}$ is the mean value of the feature *j* in the two different sample classes. $s_{1j}{}^{2}$ and $s_{2j}{}^{2}$ represent the variance of feature *j* in the two different categories of samples. The larger the value that was calculated by the Equation (2), the greater the difference in the expression of the feature *j* was in the two categories [43]. 

#### 2.2.3. Pearson Correlation Coefficient

(3) $$PC=\frac{\sum_{i=1}^{N}\left(X_{i}-\bar{X}\right)\left(Y_{i}-\bar{Y}\right)}{\sqrt{\sum_{i=1}^{N}{\left(X_{i}-\bar{X}\right)}^{2}}\sqrt{\sum_{i=1}^{N}{\left(Y_{i}-\bar{Y}\right)}^{2}}}$$

where PC is the Pearson correlation coefficient, and represents the values corresponding to the class and denotes the average of the features and categories. The greater the value of the PC, the greater was the relevance of the feature to the category [44].

#### 2.2.4. Combination of Filtered Genes

In this work, the three most commonly filters (SNR, TS, and PC) were used in order to select the genes. The three filters could obtain three orderly data sets, and each data set was sorted by value, from high to low. The top *N* genes of each data set were selected so as to integrate a new gene set.

Suppose the number of data set was S, and the number of filter was L. We obtained the gene set F, which contained the top N genes in L data sets, as follows (where we supposed that S was larger than N):

(1) Suppose $G=\left\{g_{1},g_{2},\ldots ,g_{S}\right\}$, and F=φ;
(2) Use the filter $FT_{i}$ to calculate the statistical scores and rank them, where i∈{1,2,…,L};
(3) Select the N genes with the top ranking score in each list, add N into F, and delete the N genes from G;
(4) Take the union of the L filtered lists, which consolidates the overlapping genes and reduces the size of the combined list F of the filtered genes;
(5) Repeat steps (2)–(4) until all of the top N genes are added to F and there are no duplication genes.

### 2.3. Cross-Entropy Method

The cross-entropy method [45] was a new optimization method that was proposed by Professor Reuven Y. Rubinstein in 1998 [46]. It was first used to simulate the low probability events and it was later extended in order to solve optimization problems. In recent years, the cross-entropy method was widely applied to the solution of many combinatorial optimization problems, and a solution algorithm was designed for the different application areas [47,48]. In general, the filtered gene subset F would contain a large number of redundant genes, which would affect the classification accuracy and the robustness of the classification model. Therefore, deleting the redundant genes in F played a key role in the selection of the best feature subset.

Several methods were proposed to measure the dependency of the variables [49], such as mutual information, entropy, and cross-entropy. The notion of cross-entropy was used in this work to compute the redundancy of a feature set [36,50].

The cross-entropy of f(X) and g(X), denoted by D(f(X),g(X)), is as follows:

(4) $$D\left(f\left(X\right),g\left(X\right)\right)=\sum f\left(X\right)\log\frac{f\left(X\right)}{g\left(X\right)}$$

If $f\left(X\right)=p\left(x_{1},x_{2},\ldots ,x_{n}\right)$, and $g\left(X\right)=p\left(x_{1}\right)p\left(x_{2}\right)\ldots p\left(x_{n}\right)$, the cross-entropy D(f(X),g(X)) can be written as $D_{n}$ for short.

(5) $$D_{n}=\int \ldots \int p\left(x_{1},\ldots ,x_{n}\right)\log\left[p\left(x_{1},\ldots ,x_{n}\right)/p\left(x_{1}\right)\ldots p\left(x_{n}\right)\right]$$

If $x_{1}\ldots x_{n}$ are independent, then $D_{n}=0$. Otherwise, it is as follows:

(6) $$D_{n}=\int \ldots \int p\left(x_{1},\ldots ,x_{n}\right)\log p\left(x_{1},\ldots ,x_{n}\right)-\sum_{i=1}^{n}p\left(x_{i}\right)\log p\left(x_{i}\right)$$

Let $H=-\int \ldots \int p\left(x_{1},\ldots ,x_{n}\right)\log p\left(x_{1},\ldots ,x_{n}\right)$, $H_{i}=\sum_{i=1}^{n}p\left(x_{i}\right)\log p\left(x_{i}\right)$, the above formula can be simplified as follows:

(7) $$D_{n}=-H+\sum_{i=1}^{n}H_{i}$$

Since $H_{i}\leq H\left(i=1,\ldots ,n\right)$, we have the following:

(8) $$H_{1}+H_{2}+\ldots +H_{n}\leq nH$$

For Equations (7) and (8), we have the following: $D_{n}=H_{1}+H_{2}+\ldots +H_{n}-H\leq \left(n-1\right)H$. Therefore, $D_{n}$ can be normalized as follows:

(9) $$\bar{D_{n}}=\left(H_{1}+H_{2}+\ldots +H_{n}-H\right)/\left(\left(n-1\right)H\right)$$

where $0\leq \bar{D_{n}}\leq 1$.

In general, $\bar{D_{n}}$ measures the dependency of the n variables. The larger the value of $\bar{D_{n}}$ was, the more dependent the variables were. In order to select the independent features, the threshold of independence should have been set. If the threshold of independence was T, and a feature set had $\bar{D_{n}}\leq T$, then the features in this feature set were considered to be independent.

### 2.4. Calculation of Redundancy

The gene set F, which was integrated after the filter process, was then calculated by cross-entropy, and the new gene set was obtained by removing the redundancy genes. Let $F=\left\{g_{1},g_{2},\ldots ,g_{m}\right\}$ be a feature set, which contained the *m* genes and their values of closeness with a class, we eliminated the redundant features and obtained the non-redundant feature set G as follows:

(1) Set the threshold of independence be T, and G=φ;
(2) Use $\bar{D_{n}}\left(G,g_{j}\right)$ to calculate the cross-entropy between two genes, where $g_{j}\in F$;
(3) If $\bar{D_{n}}\left(G,g_{j}\right)<T$, then $G=G\cup \left\{g_{j}\right\}$, $F=F-\left\{g_{j}\right\}$, and go to step (4);
(4) If $\bar{D_{n}}\left(G,g_{j}\right)\geq T$, then $F=F-\left\{g_{j}\right\}$, and go to step (4).
(5) Repeat (2)–(3), until F=φ.

### 2.5. Selection of Optimal Subset

To obtain the best gene set *R*, the wrapper method was used, based on the forward feature selection to select the optimal subset, with the largest classification accuracy as the criterion.

(1) Initialization R=Φ;
(2) For each $x_{i}\in G$, calculate the classification accuracy for classifier *M*;
(3) Select a subset of the genes $x_{k}$ with the highest accuracy h, $R=R\cup x_{k}$ $G=G-\left\{x_{k}\right\}$;
(4) For each $x_{i}\in G$, calculate the classification of $R\cup \left\{x_{i}\right\}$, which is referred to as $h^{\prime }$;
(5) If $h^{\prime }>h$, then $R=R\cup \left\{x_{k}\right\}$, $G=G-\left\{x_{k}\right\}$;
(6) Repeat (4)–(5), until the accuracy is 100 or G is null.

### 2.6. Flowchart of CEMFE Method

**Figure 1:**
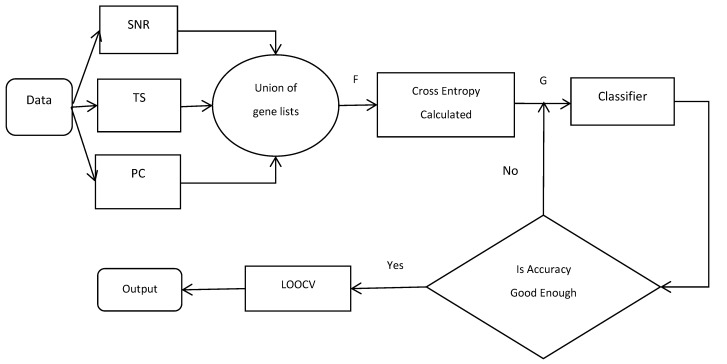
Flowchart of the cross-entropy multi-filter ensemble (CEMFE) algorithm. TS: *t*-statistic; SNR: signal-to-noise ratio; PC: Pearson correlation coefficient; LOOCV: leave one out cross validation.

In this paper, we used three of the most commonly used filters (SNR, TS, and PC) in order to select the genes from the microarray datasets and to integrate the top *N* genes in the subset of each filter, in order to form a new gene set *F*. The cross-entropy algorithm was used to get the gene set *G* from *F*. Finally, in order to get the best subset, the wrapper method was used, which was based on the forward feature selection with the accuracy as the criterion. The model that was proposed in this paper was based on the CEMFE gene selection method, shown in Figure 1:


**Our proposed algorithm can be described as follows:**
**Input:** data set S, number of filter L, number of union filtered gene P, number of genes subset (G) Q, classifier M
**Output:** optimal feature subset *R*
**For**
i=1 to L do
$S_{i}$ = use the filter $FT_{i}$ calculate the statistical scores and rank it $m_{i}$ = select m genes with top ranking score in each list
**End of For**
 F /*the union of the list of genes*/
Initialization: G=φ
**For**
j=1 to P do
Calculate $\bar{D_{n}}\left(G,x_{j}\right)$ /*For all $x_{j}\in F$*/
If $\bar{D_{n}}\left(G,x_{j}\right)<T$, $G=G\cup \left\{x_{j}\right\}$, $F=F-\left\{x_{j}\right\}$
**End of For**
**Return** G
Initialization: R=φ/*optimal feature subset*/
$x_{k}=\max Class\_Acc$, $R=R\cup \left\{x_{k}\right\}$
**For**
k=2 to Q do
newClass_Acc = calculate classification accuracy of $R\cup \left\{x_{k}\right\}$
If newClass_Acc>maxClass_Acc
maxClass_Acc=newClass_Acc, $R=R\cup \left\{x_{k}\right\}$
**End of For**
**Return**
R, maxClass_Acc


## 3. Results and Discussion

The experiments were performed on a Windows 7, 2.2 GHz 8 G personal computer All of the experiments were implemented in matlab R2015b and weka 3.8.0 [51], and three kinds of classification models, namely, the Naive Bayes, Support Vector Machine (SVM), and k-nearest neighbor, were constructed, in which the value of k was 3, and the kernel function of the SVM was set as the linear kernel function. All of the experiments were used in the K-fold cross validation method, where K was taken as 10.

### 3.1. Results on Microarray Data

The experimental datasets were first normalized using the Z-score. Then, the gene expression profile datasets were filtered, using three filters, namely, SNR, TS, and PC. Each filter produced an ordered list of genes, and the top *N* (*N* = 50) genes, with the highest rankings in each list (with a significant score on the classification), were merged to form a new list of genes. Taking the union of the three lists consolidated the overlapping genes and reduced the size of the combined list of the filtered genes. The cross-entropy algorithm was used for the redundant computing, the value of T was varied from 0.1 to 0.9, with a step size of 0.1. It was observed that the non-redundant gene sets were the same for the values between 0.4 and 0.8, therefore, we took *T* = 0.5 during the process of eliminating the redundant feature genes, (i.e., all the genes with $\bar{D_{n}}$ greater than 0.5 were rejected as dependent genes). Finally, the best feature subset was obtained using the wrapper-based forward feature selection method, and the accuracy was used as the criterion in the selection process. In learning the classification algorithm, the support vector machine (SVM) could avoid a dimensionality disaster and had better robustness [52], the training speed of Naive Bayesian (NB) was faster, and the k-nearest neighbor (KNN) was easy to implement, with no need to estimate the parameters and no need for training. In order to validate the classification model of our proposed algorithm, the three kinds of learning algorithms, including NB, SVM, and KNN, were used in order to verify their classification performance. Table 2 shows the number of feature genes and the best classification accuracy that was obtained by the different algorithms, so as to achieve the best classification accuracy. The CEMFE represents the method that was proposed in this paper; the signal–noise ration and cross-entropy (SNRCE) meant that only the signal-to-noise ratio and the cross-entropy method were used; the *t*-statistic and cross-entropy method (TSCE) meant that only the *t*-statistic and cross-entropy method were used; the Pearson correlation coefficient and cross-entropy method (PCCE) represented that only the Pearson correlation coefficient and the cross-entropy method were used.

From Table 2, we can see that for the different data sets, the different feature gene selection methods showed a different classification performance on five different classifiers. Compared with the other methods, it was shown that the accuracy of the CEMFE method was relatively high, and the number of the selected feature genes were fewer. In the colon data set, the accuracy of the CEMFE in the KNN and the NB were 96.77%, which were significantly higher than the other methods. The minimum number of genes, nine, were obtained on the NB classifier. In prostate dataset, a maximum classification accuracy of 98.04% was achieved with nine genes in the KNN classifier, using CEMFE. In the leukemia dataset, the maximum classification accuracy of 100% was achieved with 12 genes in the NB classifier, using CEMFE. The classification accuracies obtained using the CEMFE algorithm in the SVM and the KNN classifiers were also much higher than the other algorithms. In the lymphoma dataset, the maximum classification accuracy of 100% was achieved in the SVM classifier, using the CEMFE. The same classification accuracy of 98.70% was achieved in the KNN and NB classifiers. For the lung dataset, the maximum classification accuracy of 100% was achieved in the SVM and KNN classifier, using the gene that was selected by our method, CEMFE. The number of genes that were selected by CEMFE were significantly less, in comparison with the other three kinds of classification algorithms. The best result was obtained for the KNN, using only three genes. Therefore, the CEMFE algorithm that was proposed in this paper could obtain a subset of the best feature genes with a high correlation and low redundancy as a whole and could effectively improve the accuracy of the feature gene classification algorithm.

**Figure 2:**
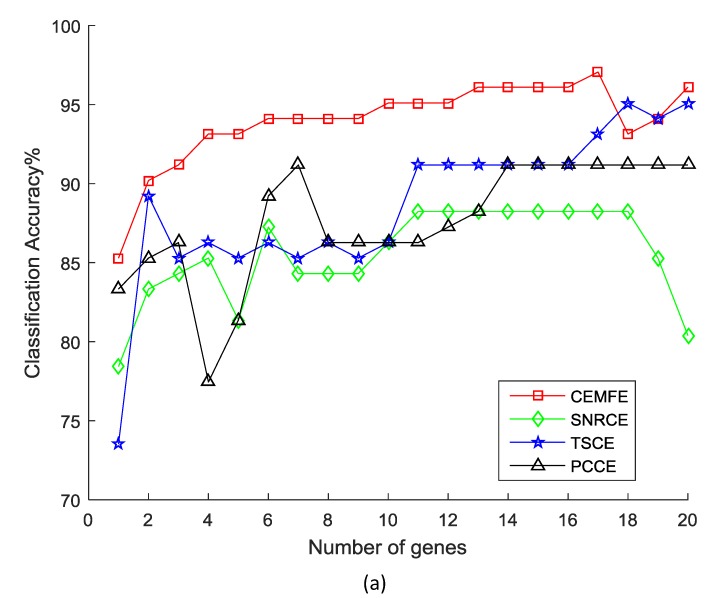

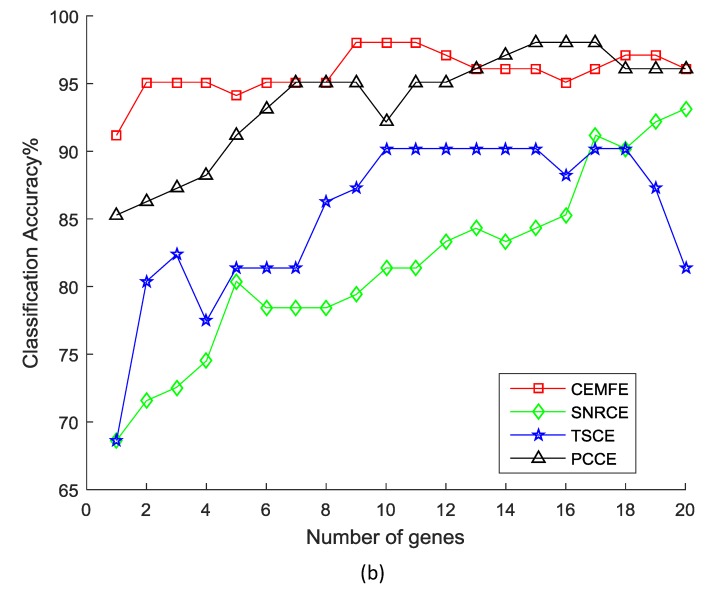

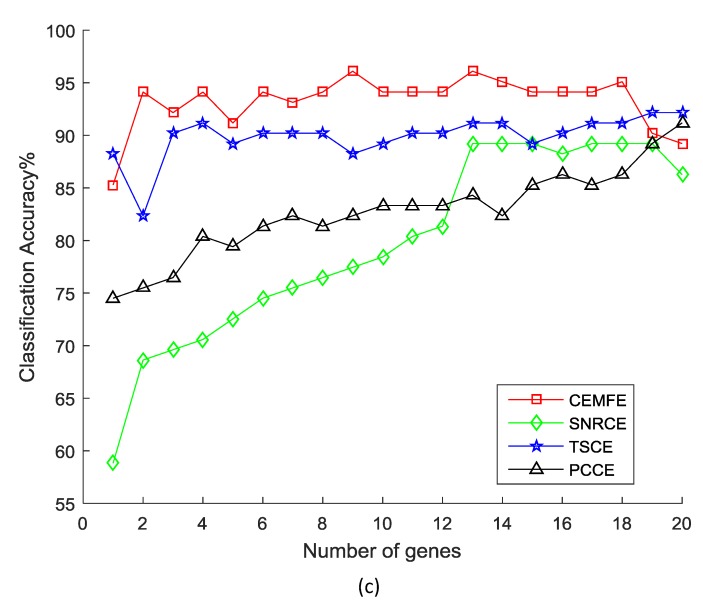
Classification accuracy vs. number of genes for the prostate dataset, using the (**a**) Support Vector Machine, (**b**) k-nearest neighbor, and (**c**) Naive Bayesian.

Figure 2 shows the results of the classification accuracy that was obtained by the different methods, as the genes were added one by one in the prostate dataset. It was observed that the classification accuracy that was obtained by our algorithm was much better than the SNRCE, TSCE, and PCCE, with the same number of genes for all of the classifiers. Similar results were also observed in the other datasets.

In order to further verify the efficiency and stability of the CEMFE classification algorithm that was proposed in this paper, we compared our methods (CEMFE) with two model-free gene selection methods, namely, mRMR [23] and FCBF [24]. The reason for choosing them was that they were typical and popular gene selection algorithms. The FCBF [24] measured the relevance between the genes using symmetric uncertainty and eliminated the irrelevant genes by virtue of an approximate Markov blanket. In mRMR [23], only those genes that might have brought more relevance to the class and less redundancy to the selected genes, at the same time, would be selected. The Naive Bayes (NB) and k-nearest-neighbor (KNN), were used in order to build the classifiers on the selected gene subsets. For KNN, K = 3 and its distance was calculated by the Euclidean formula, in our experiments. The k-fold cross-validation was used to evaluate the performance of the experiment, and K = 10.

Table 3 summarizes the best classification accuracy of NB and KNN, using three gene selectors. In the colon dataset, the accuracy of the CEMFE algorithm in both classifiers was 96.77%, which was much higher than that of the FCBF and mRMR algorithms. In the prostate dataset, the accuracy of the CEMFE algorithm in the NB classifier was slightly lower than the other two algorithms, but the classification accuracy was better for the KNN classifier, and similarly, for the leukemia dataset. In the lymphoma and lung datasets, the classification accuracy of the CEMFE algorithm on two classifiers was obviously higher than that of the FCBF and mRMR algorithms. Overall, the average classification accuracy of the CEMFE algorithm in all the five datasets was higher than the accuracy of the other two classification algorithms. In other words, the CEMFE algorithm that was proposed in this paper could obtain a highly relevant subset of the feature genes and improve the classification performance.

### 3.2. Discussion

To study the effect of varying the number of genes that were selected by each filter, we repeated all the experiments of Section 3 for each dataset, with *N* = 100 and 200, in order to ascertain whether *N* = 50 was a reasonable choice. The results for the different values of *N* are summarized in Table 4. Where the average number of genes and the average of performance, to the average of the total number of genes and the best classification accuracy that were obtained by the k-fold cross-validation on each SVM, KNN, and NB classifier are shown. For all the datasets, the best accuracy could be achieved with *N* = 50, except for lymphoma, where the accuracy had decreased from the case of *N* = 100 to that of *N* = 200, as a result of the increase of one misclassification. Theoretically, the use of a larger gene subset should have always increased the accuracy. However, the CEMFE algorithm was not guaranteed to converge to the global optimal solution, and the results of Table 4 suggested that the use of an unnecessarily large gene set might have caused the algorithm to be trapped at a local minimum, as was the case when *N* was increased from 100 to 200 for the leukemia, lymphoma, and lung data sets. Hence, for the data sets that were under consideration, *N* = 50 would be the best choice for the number of genes that were to be retained by each filter.

## 4. Conclusions

This study aimed to select an optimal subset of the features from the high dimensional and small sample gene expression datasets for the classification of cancer genes. For this purpose, we proposed a cross-entropy based multi-filter ensemble method (CEMFE), where multi-filter ensemble algorithm is used to classify the microarray data, and the smallest feature subset that is associated with cancer classification is selected. Firstly, the multi-filter is employed to select a set of relevant genes, and cross entropy is used to determine the independent genes, which provides a set of independent and relevant genes, and reduces the size of the gene set significantly. Secondly, the gene subset is selected using the wrapper method, based on the forward feature selection. Finally, the final gene subset, with the highest classification accuracy, is obtained. In the above process, the unrelated and redundant genes in the original gene set were screened, so that the obtained genes were closely related to the class labels and were independent of each other, and the best feature subset was selected, based on the criterion of the accuracy rate. The experimental results show that the method that has been proposed in this paper can not only obtain a high accuracy, but also, the number of genes that are obtained is less than that of the other methods.

## Figures and Tables

**Table 1:** Experimental datasets.

Dataset	Samples	Number of Samples		Classes
		Class 1	Class 2	
Colon	2000	40 (T)	22 (N)	2
Prostate	12,600	52 (T)	50 (N)	2
Leukemia	7129	25 (AML)	47 (ALL)	2
Lymphoma	7129	58 (DLBCL)	19 (FL)	2
Lung	12600	31 (MPM)	150 (ADCA)	2

T: tumor; DLBCL: diffuse large B-cell lymphoma; N: normal; FL: follicular lymphoma; AML: acute myeloid leukemia; ALL: acute lymphoblastic leukemia; MPM: malignant pleural mesothelioma; ADCA: adenocarcinoma.

**Table 2:** Experimental contrast of all of the kinds of algorithms on different data sets, feature gene number, and the best classification accuracy.

Dataset	Classifier	CEMFE	SNRCE	TSCE	PCCE
Colon	SVM	93.55 (23)	90.32 (7)	90.32 (18)	93.55 (33)
	KNN	96.77 (14)	88.71 (17)	83.87 (13)	90.32 (7)
	NB	96.77 (9)	88.71 (17)	85.48 (6)	91.91 (21)
Prostate	SVM	97.10 (17)	88.24 (11)	97.10 (22)	91.18 (7)
	KNN	98.04 (9)	94.12 (26)	90.20 (10)	98.04 (15)
	NB	96.10 (9)	89.22 (13)	93.14 (22)	92.16 (27)
Leukemia	SVM	97.22 (6)	95.83 (17)	90.28 (17)	95.83 (35)
	KNN	98.61 (7)	89.06 (28)	88.89 (23)	93.06 (8)
	NB	100 (12)	83.33 (26)	96.88 (19)	94.44 (33)
Lymphoma	SVM	100 (26)	88.31 (34)	96.10 (66)	84.42 (36)
	KNN	98.70 (16)	93.51 (7)	79.22 (14)	97.40 (41)
	NB	98.70 (18)	94.81 (9)	96.10 (14)	80.52 (22)
Lung	SVM	100 (4)	98.34 (24)	100 (36)	99.45 (33)
	KNN	100 (3)	100 (21)	100 (40)	100 (18)
	NB	98.90 (9)	96.13 (17)	98.90 (23)	98.90 (41)

SVM: support vector machine; KNN: k-nearest neighbor; NB: Naive Bayesian; CEMFE: cross-entropy based multi-filter ensemble; SNRCE: signal–noise ration and cross-entropy; TSCE: *t*-statistic and cross-entropy method; PCCE: Pearson correlation coefficient and cross-entropy method.

**Table 3:** The best classification accuracy of the CEMFE, FCBF, and mRMR algorithms in different data sets.

Dataset	NB			KNN		
	FCBF	mRMR	CEMFE	FCBF	mRMR	CEMFE
Colon	91.94	88.79	96.77	88.71	77.42	96.77
Prostate	97.06	98.04	96.08	97.06	97.06	98.04
Leukemia	100	100	100	100	100	98.61
Lymphoma	93.51	94.81	98.70	93.51	97.40	98.70
Lung	86.67	99.13	100	83.33	96.13	98.90
Average	93.84	96.15	98.31	92.52	93.60	98.20

mRMR: minimum redundancy maximum relevance; FCBF: fast correlation based filter.

**Table 4:** Classification accuracies variation on different number of genes selected by each filter.

N/Data Set		Colon	Prostate	Leukemia	Lymphoma	Lung
50	Avg. no. of genes	15.3	12.3	8.3	20	5.3
	Avg. performance	95.70	97.08	98.61	99.13	99.63
100	Avg. no. of genes	10.6	12.3	11.6	20	5.3
	Avg. performance	93.55	95.93	93.06	100	99.63
200	Avg. no. of genes	10.6	12.3	13.3	22.3	3.6
	Avg. performance	91.94	95.93	90.28	98.70	98.90

N: number of genes selected; Avg: average; no: number.
